# DERL2 (derlin 2) stabilizes BAG6 (BAG cochaperone 6) in chemotherapy resistance of cholangiocarcinoma

**DOI:** 10.1007/s13105-023-00986-w

**Published:** 2023-10-10

**Authors:** Luzheng Liu, Jincai Wu, Yanggang Yan, Shoucai Cheng, Shuyong Yu, Yong Wang

**Affiliations:** 1 Department of Interventional Radiology and Vascular Surgery, The Second Affiliated Hospital of Hainan Medical University, Hainan, 570311 China; 2 Department of Hepatobiliary and Pancreatic Surgery, Hainan General Hospital, Hainan Affiliated Hospital of Hainan Medical University, Hainan, 570311 China; 3 Department of Gastrointestinal Surgery, Hainan Cancer Hospital, Hainan, 570312 China

**Keywords:** Cholangiocarcinoma, Chemosensitivity, DERL2, BAG6

## Abstract

DERL2 (derlin 2) is a critical component of the endoplasmic reticulum quality control pathway system whose mutations play an important role in carcinogenesis, including cholangiocarcinoma (CHOL). However, its role and its underlying mechanism have yet to be elucidated. Herein, we revealed that *DERL2* was highly expressed in CHOL and considered as an independent prognostic indicator for inferior survival in CHOL. *DERL2* ectopically expressed in CHOL cells promoted cell proliferation and colony formation rates, and depleting *DERL2* in CHOL cells curbed tumor growth in vitro and in vivo. More interestingly, the knockout of *DERL2* augmented the growth-inhibitory effect of gemcitabine chemotherapy on CHOL cells by inducing cell apoptosis. Mechanistically, we discovered that DERL2 interacted with BAG6 (BAG cochaperone 6), thereby extending its half-life and reinforcing the oncogenic role of BAG6 in CHOL progression.

## Introduction

Cholangiocarcinoma (CHOL) is a heterogeneous epithelial cell tumor that represents approximately 10 to 20% of hepatic cancer and 2% of all cancers [1]. It mainly arises from the peripheral locations within the intrahepatic bile ducts, with cholangiocyte differentiation features [2]. Surgery resection combined with traditional therapy is the first option to treat CHOL patients [3, 4]. However, just a few CHOL patients respond well, resulting in favorable long-term prognoses [5]. The risk of neoplastic development is potentiated and exacerbated by chronic inflammation, infections, and cholestasis [6]. Additionally, genetic disorders sustain tumor cell proliferation, migration, and survival, consequently being identified as risk factors [7, 8]. Hence, there is a paramount need to explore novel therapeutic targets for CHOL patients.

Persistent endoplasmic reticulum (ER) stress is regarded as a friend or a foe of tumorigenesis and cancer development, exerting context-dependent effects on tumor cell growth or cell death [9–13]. The endoplasmic reticulum (ER), an essential organelle in eukaryotic cells, assumes critical functions in protein synthesis, folding, and transportation. It intricately coordinates protein folding and export processes [14–16]. This protein ER quality control system is responsible for maintaining adequate ER proteostasis via endoplasmic reticulum-associated degradation (ERAD) machinery [17–19]. This intricate proteostasis is indispensable to determining cellular function and behavior [20–22]. However, when external stressors disrupt this delicate equilibrium, misfolded proteins accumulate within the ER, leading to ER stress, which contributes to the survival and proliferation of cancer cells [23, 24]. Additionally, the ER plays a crucial role in lipid metabolism, and calcium signaling, as well as cancer angiogenesis and invasion. Among the membrane protein family known as Derlins, which form dislocation pores through transmembrane domain oligomerization and facilitate ER degradation of misfolded glycoproteins, three highly homologous members have been identified: Derlin1 (derlin 1), Derlin2 (derlin 2), and Derlin3 (derlin 3). Recent studies have suggested amplification of the DERL1 protein is involved in cell behavior and functionality of breast cancer [25], colon cancer [26], and bladder tumors [27]. *DERL3* silence confers the cell’s unlimited proliferation potency and drives the progression of breast cancer [28], lung adenocarcinoma [29], and cervical cancer [30]. DERL2, an ER membrane-associated and luminal protein characterized by three predicted loops, has been shown to cause perinatal lethality in whole-body *DERL2* deletion mice, with the surviving mice developing skeletal dysplasia due to abnormal accumulation of collagen matrix proteins within the ER lumen [31]. In chronic lymphocytic leukemia, preclinical and clinical evidence has shown the amplification of *DERL2* mRNA levels [32]. However, its role and mechanism in cancers are hardly disclosed.

In this study, we employed RNA-sequencing data from TCGA (The Cancer Genome Atlas) to investigate the relationship between the expression of the Derline protein family and the progression of CHOL, shedding light on the potential oncoprotein role of DERL2 in CHOL. Additionally, we elucidated the influence of DERL2 on CHOL growth and uncovered the therapeutic potential of targeting the DERL2-mediated signaling axis through preclinical investigations.

## Methods

### Bioinformatics analysis

#### DERL2 expression in TCGA pan-cancers and its prognostic implication

The RNA sequencing data of pan-cancer were collected from TCGA normal and TCGA tumors (https://portal.gdc.cancer.gov/) [33]. After transformation to log2, the expression data were analyzed by the Mann-Whitney *U* test and plotted on the “ggplot” package of the R language. The clinical information of the TCGA pan-cancer cohort was used to evaluate the impact of *DERL2* expression on the clinical outcome of CHOL patients.

#### Gene set enrichment analysis

An analysis of gene set enrichment analysis (GSEA) on the website (https://www.broadlnstitute.org/gsea/) [34] was conducted to verify the signaling cascades related to DERL2.

#### Immune infiltrate correlation analysis using Tumor Immune Single-cell Hub database

A correlation analysis with immune checkpoints was performed via Tumor Immune Single-cell Hub (TISCH) online database to examine the impact of different variants of DERL2 on tumor immune infiltration (immune cell and immune checkpoint molecules) [35].

#### Analyzing DERL2 expression in CHOL cells

*DERL2* mRNA data were retrieved from the Cancer Cell Line Encyclopedia (CCLE) website (http://www.broadinstitute.org/ccle) [36]. Its expression in a panel of CHOL cells was visualized and plotted.

#### Drug sensitivity analysis

Using the R package “oncopredict” based on the Genomics of Drug Sensitivity in Cancer database (GDSC) [37], the sensitivity score (half-maximal inhibitory concentration (IC50)) of all drugs presented in the Genomic dataset was calculated to examine the potential for treating *DERL2*-low and *DERL2*-high CHOL patients

#### Cells and plasmids

Cholangiocarcinoma cell line QBC939, RBE, HUCCT1, HCCC9810, and human intra-hepatic biliary epithelial cells (HiBECs) were obtained from Fenghuishengwu Science & Technology Co., Ltd., Hunan, China. QBC939, HUCCT1, and HCCC9810 cells were grown in Dulbecco’s modified Eagle’s medium (DMEM, Sigma, USA), RBE cells and HiBECs were cultured in Roswell Park Memorial Institute 1640 medium (RPMI-1640, ThermoFisher USA). Both mediums contained 10% FBS (fetal bovine serum, Thermofisher, China) and 1% penicillin/streptomycin (ThermoFisher, USA). In addition, both cells were in a CO_2_ incubator (5% CO_2_, 95% air, 37°C). Plasmid transfections were performed with cationic liposomes (LipofectAMINE Plus; Life Technologies).

The constructed vectors carrying *DERL2-Myc* and *BAG6* (*BAG cochaperone 6*)*-HA* were generated using PCR and cloned into pCDNA5/FRT/TO-Myc (Thermofisher, USA) or pCDNA5/FRT/TO-HA (Thermofisher, USA), respectively. To overexpress DERL2 in QBC939 cells, the cDNA sequence of *DERL2* was amplified from QBC939 cells and inserted into pCDNA5 vectors (Thermofisher, USA). The produced vectors were transfected into QBC939 cells. The corresponding empty vectors were transfected into QBC939 cells and served as the controls.

### Western blot

Cells were lysed in RIPA buffer (Solarbio, China) by sonication. After centrifugation at 15,000 *g* for 10 min, the protein concentration of the supernatant was determined using BCA Protein Assay (BCA) Kit (Beyotime, China). Twenty micrograms of proteins was mixed with 5× loading buffer and boiled at 95°C for 5 min before being loaded into 12% polyacrylamide gel for electrophoresis. After SDS-PAGE, proteins were transferred onto PVDF membranes using a blotting assembly sandwich system. Following blocking in 15 ml of blocking solution for 1 h at room temperature, the membranes were incubated with the primary antibody at 1:2,000 in 1× TBST (Tris-buffered saline containing 0.2% Tween-20) at 4 °C. The next day, the secondary antibody at a dilution of 1:3000 to 1:10,000 was added and incubated at room temperature for 2 h. The blots were developed using ECL Western Blotting Detection Kit (Amylet Scientific, China). Image J software was used to analyze the protein levels. Anti-Flag antibody (Cat#BN20611, Biorigin, China), Anti-HA antibody (Cat#DE0612, Biorigin, China), Anti-GAPDH antibody (Cat#DE0621, Biorigin, China), anti-DERL2 antibody (Cat# NDC-ASJ-CJRQXJ-50, Amylet scientific, China), anti-BAG6 antibody (Cat#K112847P, Solarbio, China), Anti-PARP1 antibody (Cat#ab191217, Abcam, USA), Anti-Cleaved PARP1 antibody (Cat#ab32064, Abcam, USA), Anti-Capase-3 antibody (Cat#ab32351, Abcam, USA), Anti-Cleaved Capase-3 antibody (Cat#E83-77, Abcam, USA), Anti-GAPDH antibody (Cat#ab9485, USA), and Anti-beta Actin antibody (Cat#ab8227, USA) were used in western blot analysis.

### RT-qPCR

RNAs were isolated with a Trizol-chloroform method in a ratio 1:5 (chloroform: Trizol) (TRIzol, Invitrogen, USA) [38]. Using a NanoDrop Spectrophotometer, RNA concentration was determined. Reverse transcribe RNA into cDNA was performed using SuperScript™ IV One-Step RT-PCR system (Thermo Fisher Scientific, Inc.). The levels of gene transcripts were assessed using Luna Universal qPCR Master Mix (NEB). 2^−ΔΔdCT^ relative quantification method was used. The *DERL2* primer is as follows: Forward primer 5′ CGCCGCCGTGCAGTT 3′, Reverse primer 5′AAAATCCCATGAGCACCCAGG 3′.

### Construction of DERL2 knockout CHOL cells

Using the CRISPR (http://crispor.tefor.net/), two sgRNAs targeting *DERL2* Exon5 and Exon6 were designed and then synthesized by Tianyi Huiyuan Biotechnology Co. Ltd. China. Each of the two complementary sgRNA sequences was annealed at 95 °C, resulting in the formation of double-stranded duplexes. Subsequently, the complexes underwent restriction digestion and were inserted into the pLentiCRISPRv2 vector to generate the Lentiv2-sgRNA vector. LentiV2-sgRNA or Lentiv2 vectors were packaged in 293T cells with psPAX2 and pMD2.G. Forty-eight hours later, the cell culture was filtered with 0.22-μm filters (Anotop, Whatman). The medium, containing virus particles, was used to infect CHOL cells for another 48 h. The infected cells were selected with 3 μg/ml puromycin (Sigma, USA). After 2 weeks, positive clones were collected and amplified, followed by confirmation through Sanger sequencing and western blot analysis. The sgRNA sequences were listed below 5′ GAGCTTAGTTTTCTTGGGCCAGG3′, 5 ′GTATTTCCCAATCAACCTGGTGG3′.

### CCK8 assays

A total of 1000 cells were seeded in each well of a 96-well plate. After 24 h, 48 h, or 72 h, 100 μL/well solution of CCK8 was added and incubated for 1 h. The plates were subsequently analyzed using a microplate reader at a wavelength of 450 nm.

To evaluate the impact of different drugs on CHOL cell proliferation, varying concentrations of Gemcitabine (0, 1, 2, 5, or 5 ng) were added to the 96-well plates and maintained for 48 h. Following the incubation period, cell proliferation was assessed using CCK8 assays.

### Colony formation assays

In a 12-well plate, 400 cells were seeded onto and grown for 14 days. Subsequently, the cells were fixed and stained in 1 mL in 100% ethanol containing 0.25% crystal violet. The staining was allowed to stand for 20 min and then the cell colony was counted.

### Apoptosis assay

Annexin V-FITC/PI Apoptosis Detection Kit (enzyme, China) was applied to determine the QBC939 cell apoptosis when *DERL2* knockout or not. In short, the transfected QBC939 cells (KO-1 and KO-2) and wild-type (WT) QBC939 cells were plated on 6-well plates. Forty-eight hours later, the cells were harvested and trypsinized without EDTA. Subsequently, 100 μl of 1× binding buffer was added to resuspend the cells, followed by the addition of propidium iodide (PI; 5 μl) and Annexin V-FITC (5 μl). The staining reaction was conducted in the dark at room temperature, and after 10 min, the cells were treated with 200 μl of 1× binding buffer. The apoptotic cells were counted using a BD FACScan™ flow cytometer (BD Biosciences) within 1 h, and the data was read using BD FACSuite™ (BD Biosciences).

### Cell cycle analysis

The cell cycles were examined using Cell Cycle and Apoptosis Analysis Kit (PI staining, Medchemexpress, China). The transfected cells (2×10^5^ cells/ml) were plated on 6-well plates. Two days later, the cells were centrifuged, harvested, and rinsed with PBS following fixation with 70% pre-chilled ethanol at 4 °C. On the following day, the ethanol was removed by centrifugation, and the cells were further washed with PBS and resuspended in a pre-prepared PI working solution (PI/RNase A ratio, 1:9). After incubation with the PI working solution at room temperature for 1 h, the apoptotic cells were quantified using a BD FACScan flow cytometer.

### In vivo assays

Thirty BALB/c nude mice (4–5 weeks of age, 16–20 g) were purchased from the Wuhan University Center for Animal Experiment/Animal Biosafety Level III laboratory (ABSL-III lab) of Wuhan University (Wuhan, Hubei, China). The study was approved by the institutional ethics Committee of Hainan Medical University (No.GKJ190015). The mice were assigned into three groups (*n*=10): wild-type group (WT), KO-1, and KO-2 groups. Subcutaneous injections of the indicated QBC939 cells were conducted on the back of mice. The tumor size was measured every 5 days. The tumor weight was recorded following euthanasia with CO_2_. Tumor volume=tumor length×tumor width^2^/2.

### Co-immunoprecipitation assays

For coimmunoprecipitation assays, we prepared QBC939 cells that were transfected with Flag-*DERL2* vectors, as well as 293T cells that were cotransfected with HA-*BAG6* and Flag-*DERL2* vectors. Following centrifugation at 16,000 *g* for 15 min at 4 °C using a microcentrifuge, the protein concentration was determined using the Pierce BCA Protein Assay Kit. Subsequently, 50–100 μg of total cell lysate, along with the recommended amount of antibody, were mixed on ice and incubated overnight at 4 °C with gentle rotation. The resuspended protein A/G PLUS-Agarose (Santa Cruz, Cat. #sc-2003) was added and incubated at 4 °C for 5~6 h. After centrifugation at 2500 *g* for 5 min at 4 °C, the immunoprecipitates were subjected to western blot analysis.

### Protein half-life analysis

Two days post-transfection, cells were exposed to different concentrations of CHX (Cycloheximide, MKBio, China) at the indicated time points. Western blot analysis was carried out to detect the DERL2 or BAG6 expression.

### Statistical analysis

Statistical significance was determined at a significance level of *P*<0.05. The data are presented as mean ± standard error of the mean (SEM). Statistical analysis was performed using Prism 8. For comparisons involving more than two groups, one-way ANOVA followed by the Tukey test was employed. The unpaired Student *t*-test was utilized for comparisons between the two groups.

## Results

### Analysis of the Derline protein in the CHOL malignancy

**Fig. 1:**
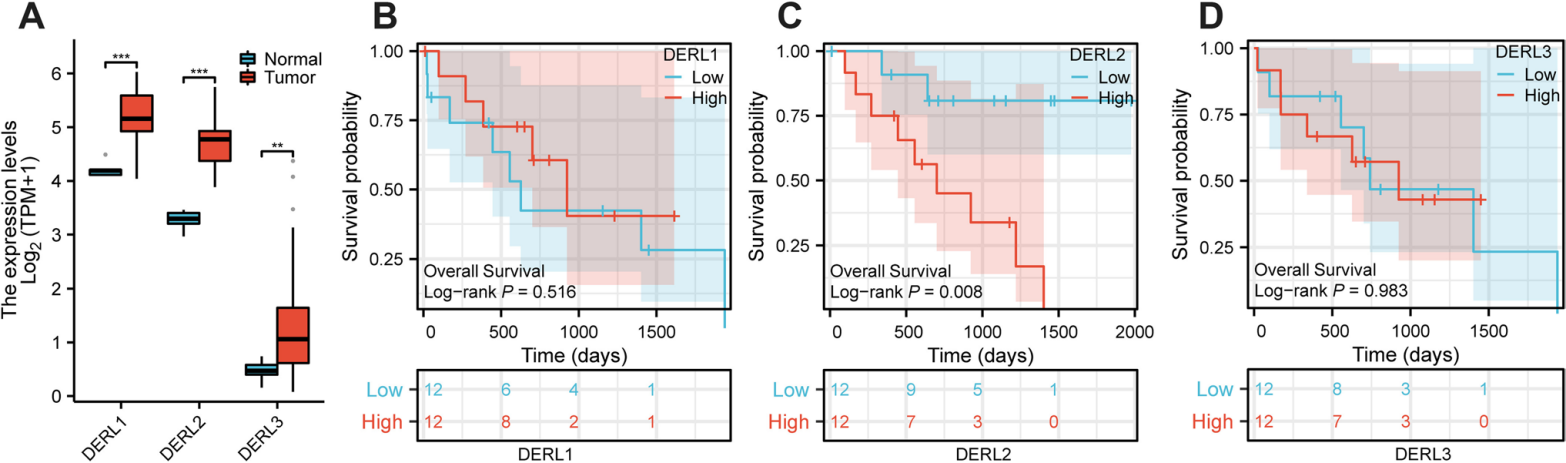
Expression and prognosis of the Derline protein in CHOL malignancy. **A** The expression of three members of the Derline family in the TCGA CHOL cohort. **B** Overall survival by *DERL1 e*xpression. **C** Overall survival by *DERL2* expression. **D** Overall survival by *DERL3* expression. Significance: **p < 0.01, ***p < 0.001

Considering the significance of Derline protein in CHOL malignancy, we analyzed to explore their expression profile and prognostic implications in patients with CHOL, utilizing data from the TCGA CHOL cohort. Figure 1 A illustrates that the expression levels of the three *Derline* genes were notably elevated in CHOL tissues compared to their corresponding normal tissues. While the expression of *DERL1* and *DERL3* did not exhibit a significant impact on the overall survival of CHOL patients (*P*=0.516, *P*=0.983, respectively), patients with high expression of *DERL2* demonstrated a worse overall survival outcome in comparison to those with low *DERL2* expression (*P*=0.008) (Fig. 1B–C). These findings piqued our interest in investigating the functional role of DERL2 in CHOL patients.

**Fig. 2:**
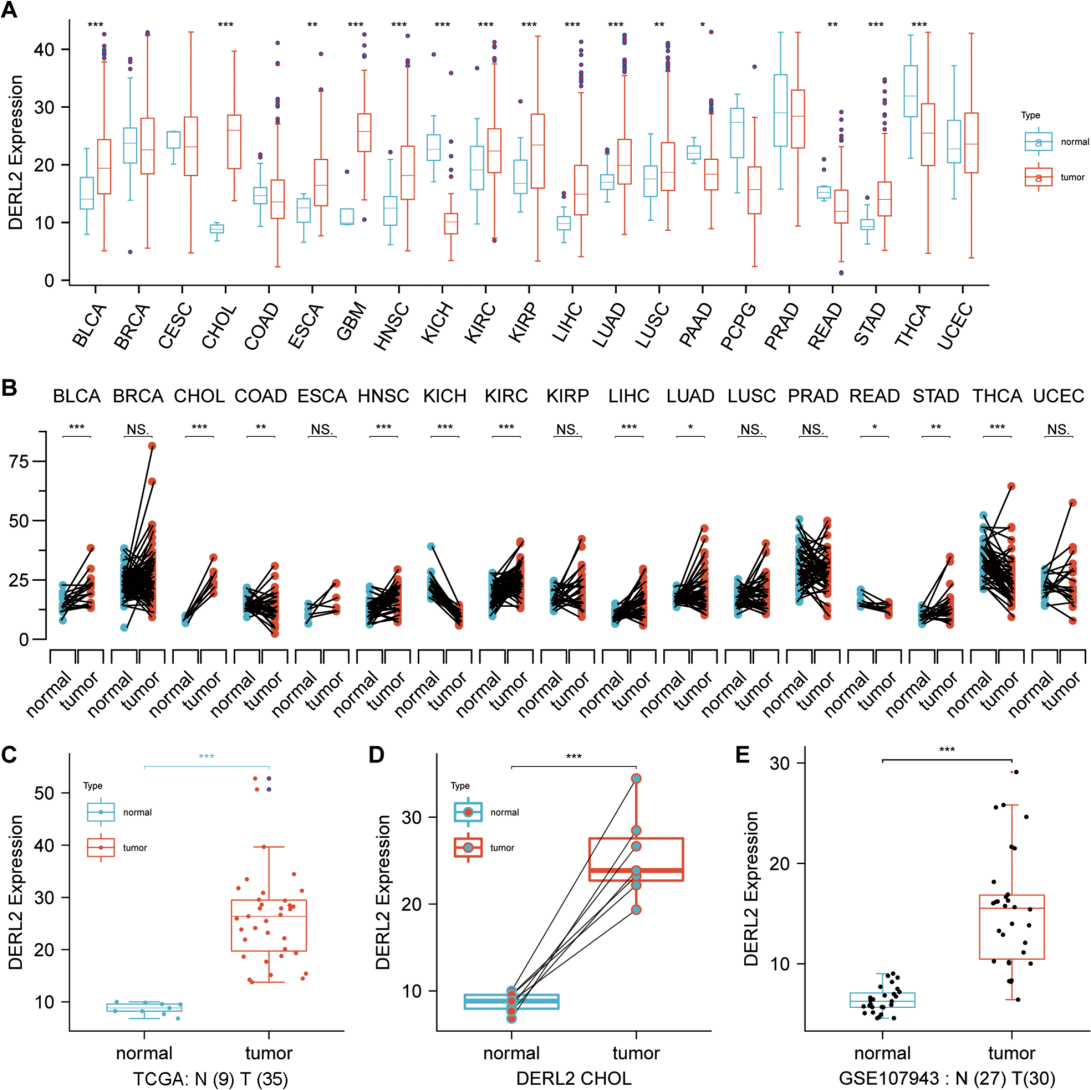
Pan-cancer expression landscape of DERL2. **A** Comparison of *DERL2* expression in the TCGA tumor tissue and normal tissues. **B** Comparison of *DERL2* expression in the TCGA tumor tissue and the paired TCGA normal tissues. **C** Comparison of *DERL2* expression in the TCGA CHOL tumor tissue. **D** Comparison of *DERL2* expression in the TCGA CHOL tumor tissue and the paired TCGA normal tissues. **E** Analysis of *DERL2* expression in the CHOL based on GEO database GSE107943. BLCA, bladder urothelial carcinoma; BRCA, breast invasive carcinoma; CESC, cervical squamous cell carcinoma and endocervical adenocarcinoma; COAD, colon adenocarcinoma; ESCA, cervical squamous cell carcinoma and endocervical adenocarcinoma; GBM, glioblastoma multiforme; HNSC, head and neck squamous cell carcinoma; KICH, kidney chromophobe; KIRC, kidney renal clear cell carcinoma; KIRP, kidney renal papillary cell carcinoma; LIHC, liver hepatocellular carcinoma; LUAD, lung adenocarcinoma; LUSC, lung squamous cell carcinoma; PAAD, pancreatic adenocarcinoma; PCPG, pheochromocytoma and paraganglioma; PRAD, prostate adenocarcinoma; READ, rectum adenocarcinoma; STAD, stomach adenocarcinoma; THCA, thyroid carcinoma; UCEC, uterine corpus endometrial carcinoma; CHOL, cholangiocarcinoma. Significance: *p<0.05, **p < 0.01, ***p < 0.001

**Fig. 3:**
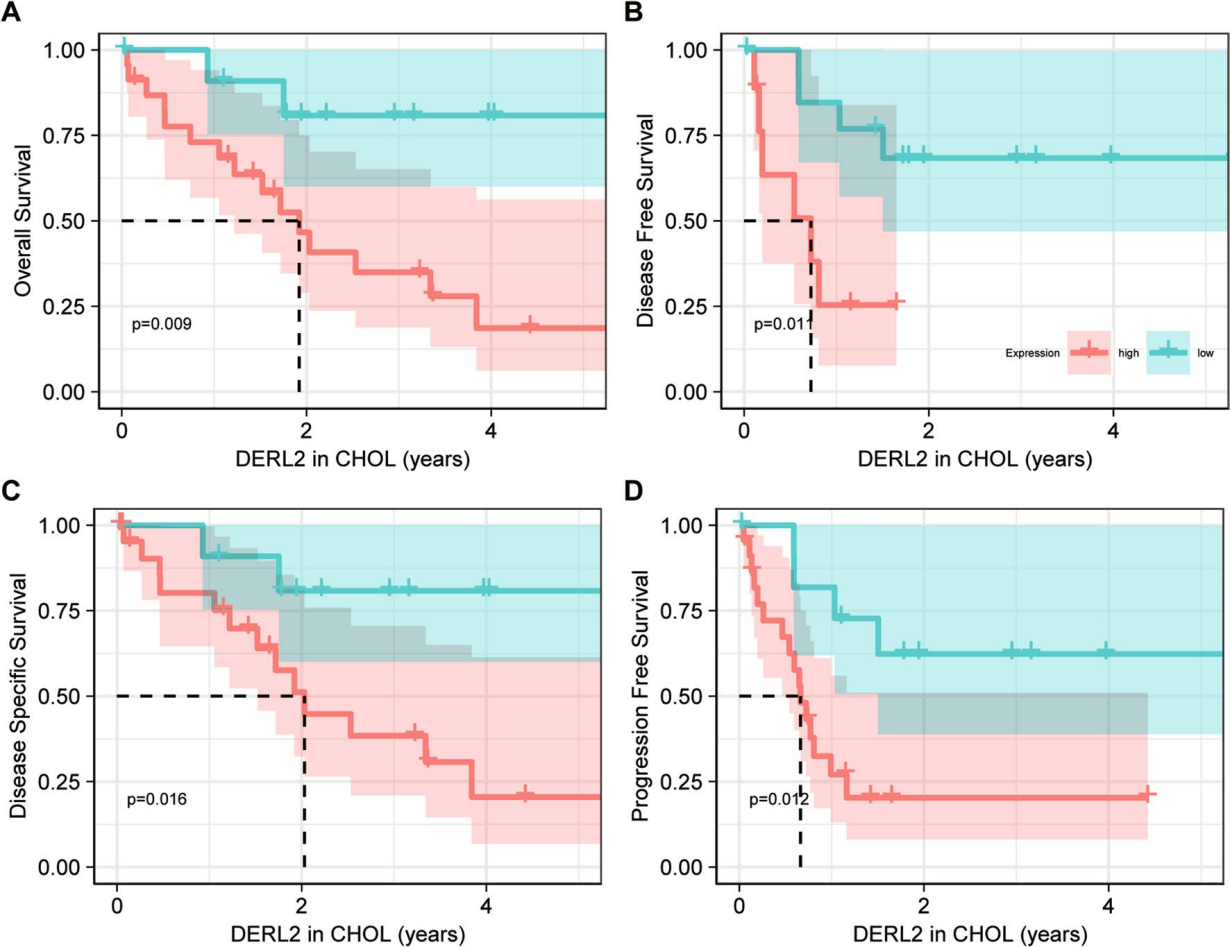
Prognostic significance of DERL2 in CHOL. **A** Overall survival. **B** Disease-free survival. **C** Disease-specific survival. **D** Progression-free survival

**Fig. 4:**
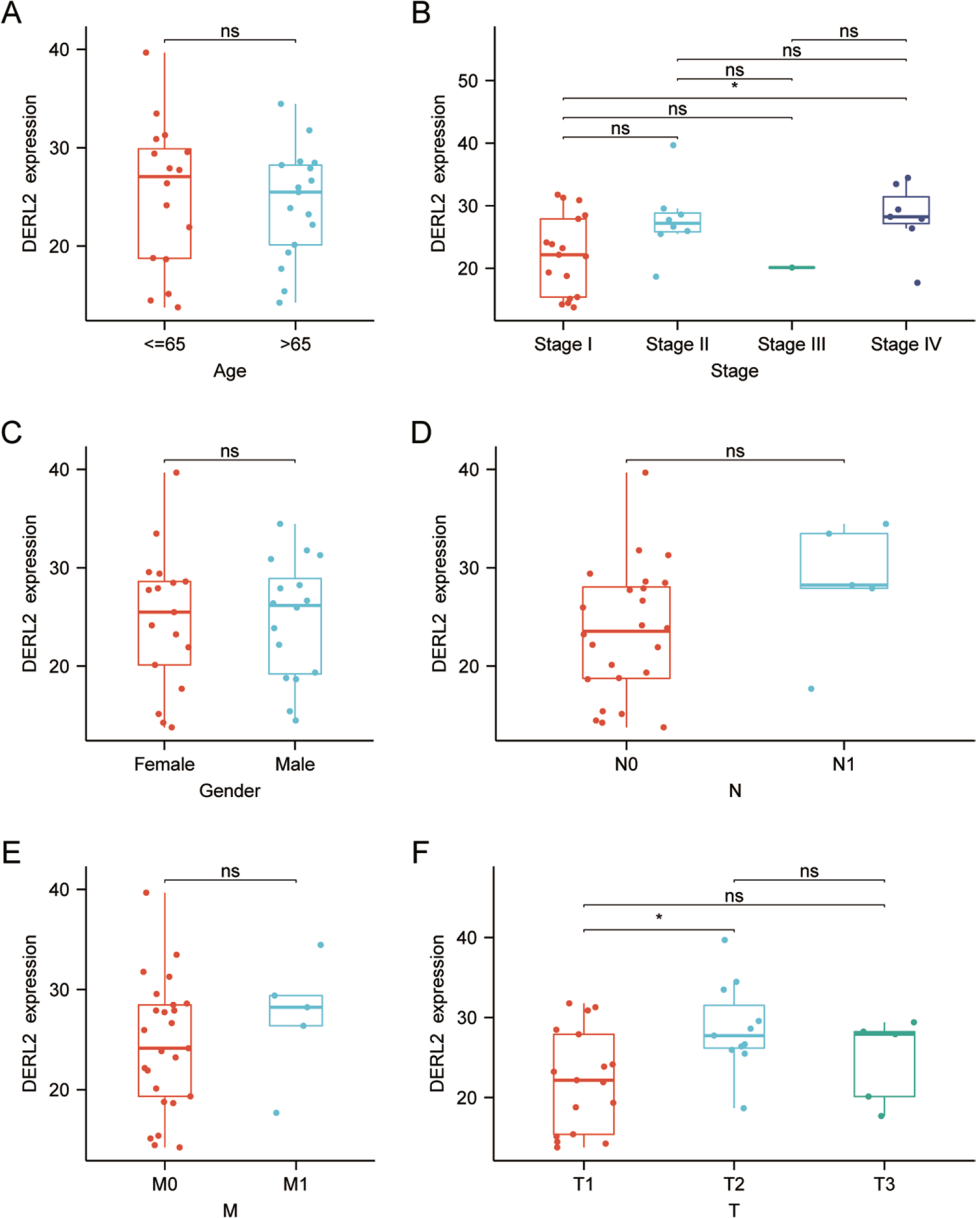
Comparison of *DERL2* expression between CHOL patients with ≤65 years and > 65-year group (**A**), among I–IV stage groups (**B**), between female and male groups (**C**), between lymph node metastasis N0 and N1 groups, between distant metastasis M0 and M1 group (**E**), among patients with tumor 1 (T1) stage, tumor 2 (T2) stage, tumor 3 (T3) stage (F)

Subsequent bioinformatics analysis was performed to elucidate the pan-cancer expression landscape of *DERL2*, as the availability of normal tissues and paired tumor tissues was limited for differential expression analysis across various cancers. Utilizing the TCGA pan-cancer cohort’s normal/tumor data, we observed the prevalence of *DERL2* mRNA expression across a wide range of cancer types, including CHOL (Fig. 2A). Consistently, a comparison of *DERL2* expression in TCGA tumors and normal tissues revealed elevated mRNA levels of *DERL2* in pan-cancer samples (Fig. 2B–D). To further analyze its expression pattern, we retrieved data from the GEO database (GSE107943), confirming the high expression of *DERL2* in CHOL tissues (Fig. 2E). To assess the impact of DERL2 on the clinical outcome of CHOL patients, we analyzed the TCGA-CHOL cohort. Notably, DERL2 amplification was associated with adverse prognosis across multiple survival measures, including overall survival, disease-free survival, disease-specific survival, and progression-free survival (Fig. 3). However, no significant differences were observed in *DERL2* expression among different patient subgroups categorized by gender, age, and TNM stage (Fig. 4).

**Fig. 5:**
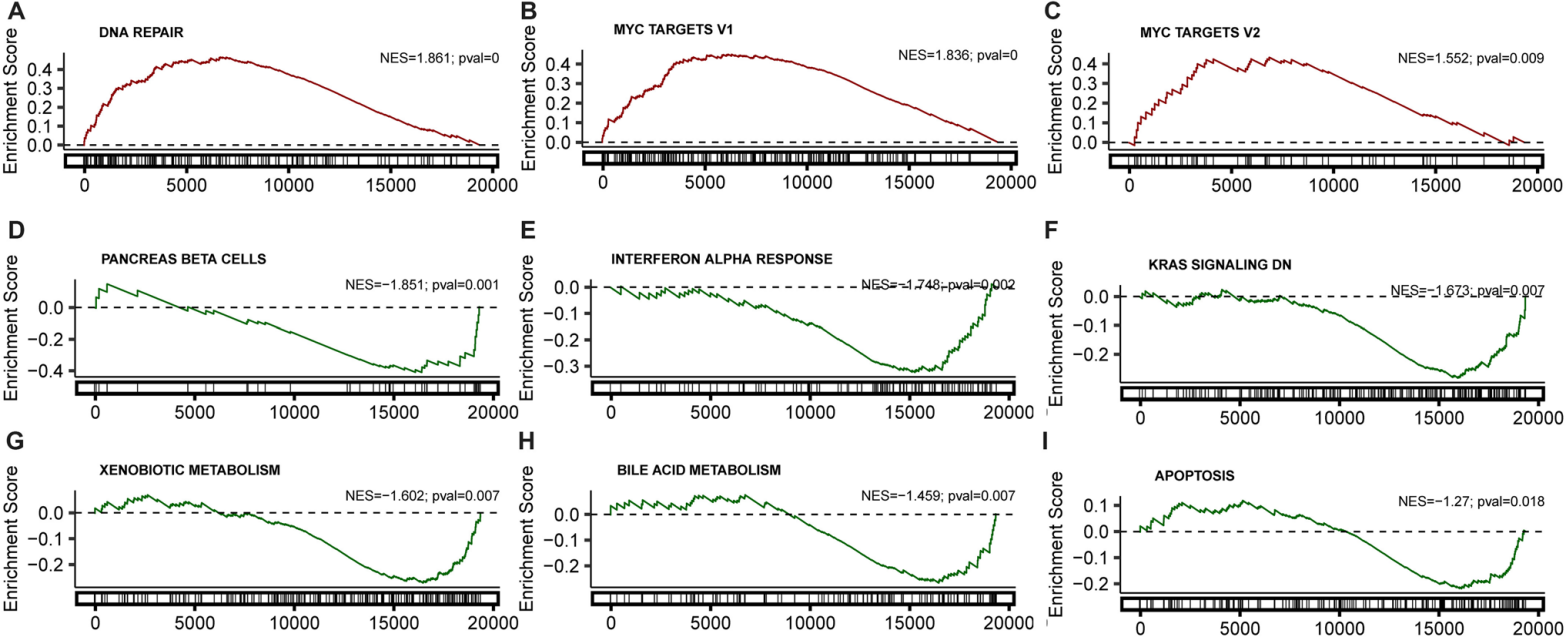
Identification of *DERL2*-associated signaling pathways. **A** GSEA DNA repair-associated genes. **B** GSEA Myc target gene set. **C** GSEA Myc target gene set. **D** GSEA Kras signaling pathway. **F** GSEA apoptosis–related gene set. **G** GSEA interferon aloha response. **H** GSEA bile acid metabolism. **I** GSEA pancreas beta cells. **K** GSEA xenobiotic metabolism

To unravel the signaling mechanism underlying the role of DERL2 in CHOL progression, we conducted GSEA using transcriptome data from the TCGA-CHOL cohort. Our analysis revealed a selective positive enrichment of gene sets associated with DNA repair, Myc targets, and Myc targets V2, indicating their potential involvement in DERL2-mediated phenotypes (Fig. 5).

**Fig. 6:**
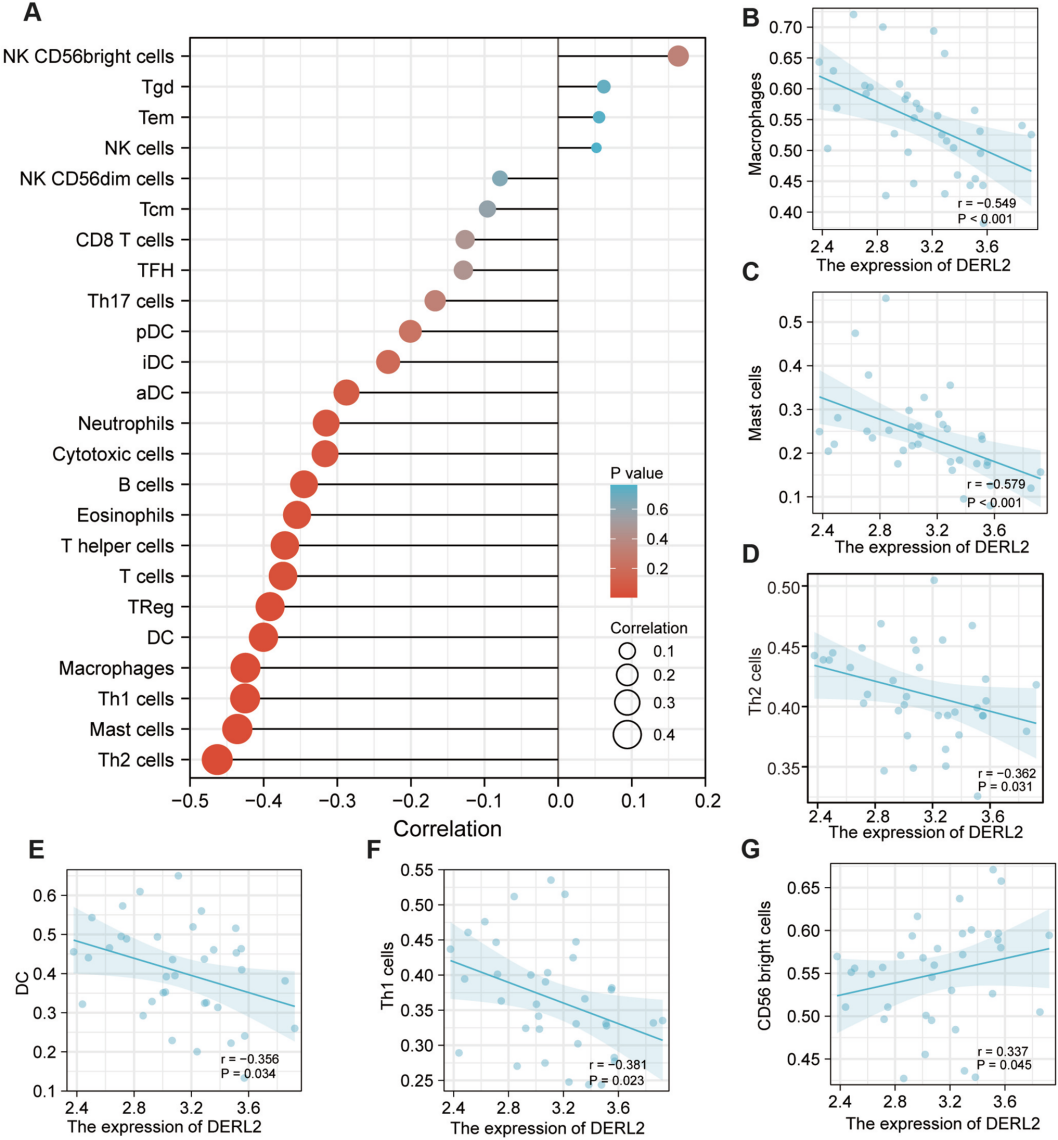
DERL2 expression correlates with immune cell infiltration in CHOL. Analyses of the correlation of DERL2 mRNA levels with immune cell infiltration in CHOL using the immune microenvironment on ISCH (http://tisch.comp-genomics.org), **A** lollipop diagram showing the correlation of the expression of DERL2 mRNA levels with the immune cell infiltration. DERL2 mRNA levels negatively or positively correlated with infiltration of **B** macrophages, **C** mast cells, **D** Th2 cells, **E** CD, **F** Th1 cells, and **G** DC56 right cells

DERL2, a crucial factor in ER-associated protein degradation (ERAD) pathways known for its involvement in host innate immunity [39], prompted us to investigate its impact on the immune microenvironment using samples from the TCGA-CHOL cohort. To explore the influence of *DERL2* expression on the immune microenvironment, we utilized samples from the TCGA-CHOL cohort and conducted the analysis using the TISCH platform. The resulting lollipop diagram (Fig. 6A) revealed noteworthy correlations between *DERL2* mRNA levels and various immune cell populations. Specifically, *DERL2* expression exhibited negative associations with macrophages (Fig. 6B), mast cells (Fig. 6C), type 2 T helper cells (Th2) (Fig. 6D), Th1 cells (Fig. 6E), and CD56 bright cells (Fig. 6F). Additionally, a positive correlation between *DERL2* and CD cells was observed (Fig. 6G). These findings shed light on the potential involvement of *DERL2* in shaping the immune landscape within the tumor microenvironment.

### DERL2 influences the CHOL cell proliferation

**Fig. 7:**
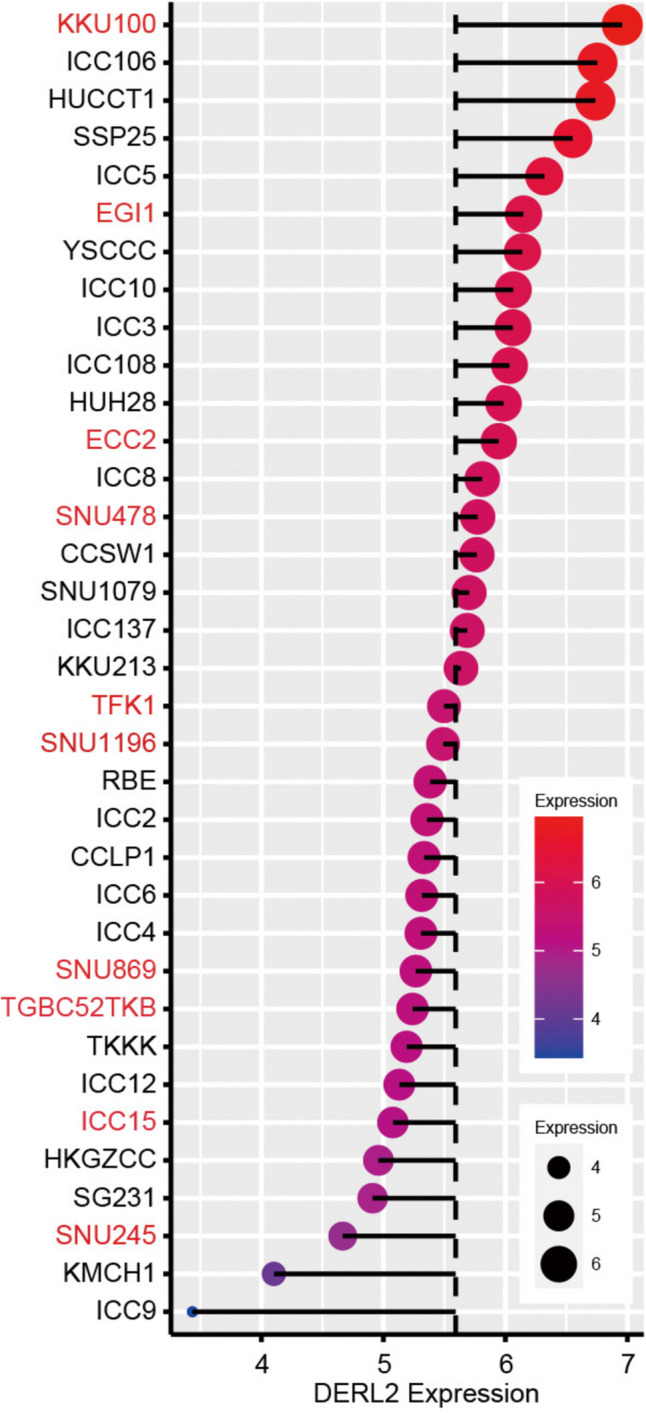
Comprehensive cell line encyclopedia (CCLE) data analysis of DERL2 transcription across a panel of CHOL cells

**Fig. 8:**
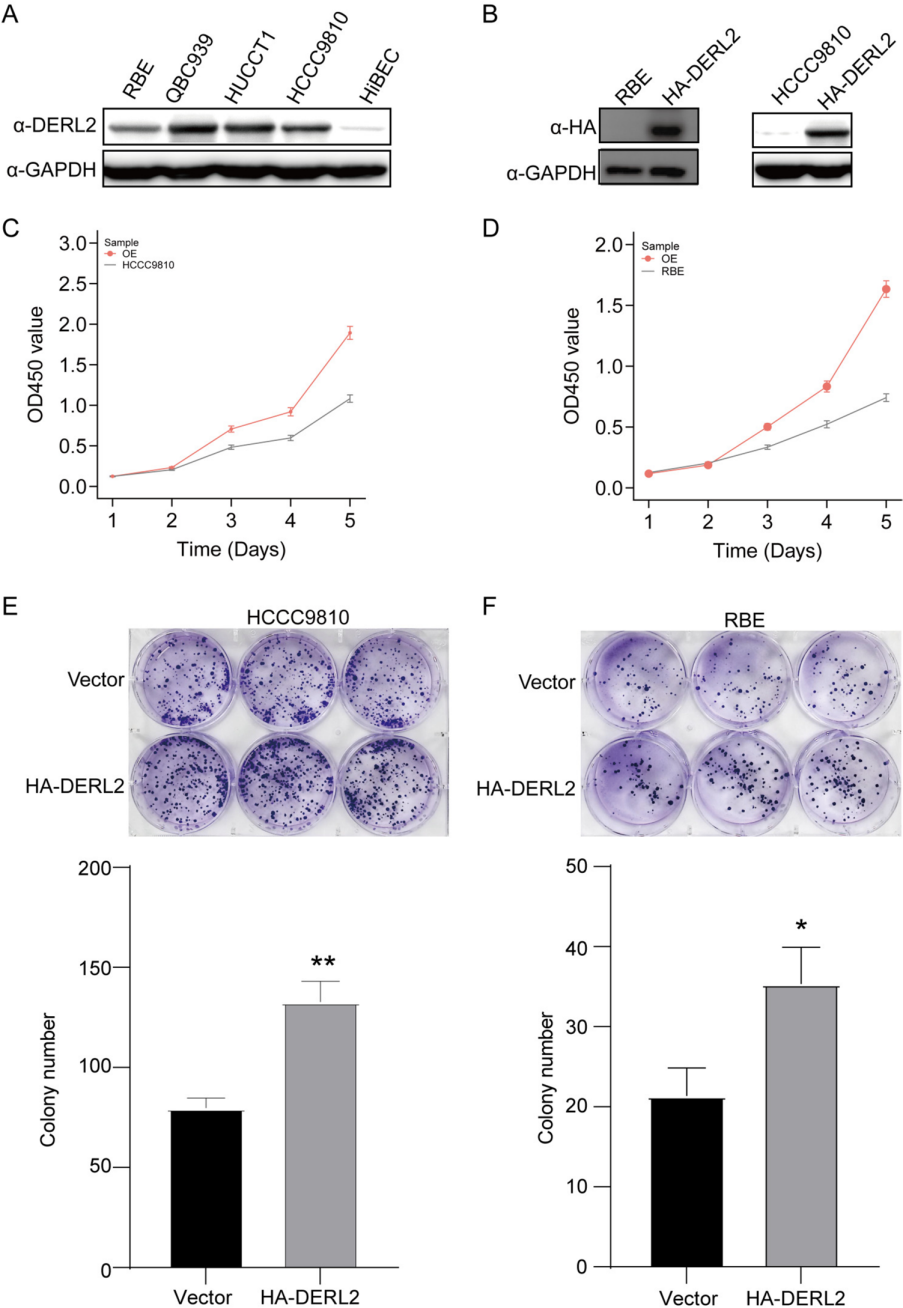
Overexpressing DERL2 boosts the CHOL cell proliferation. **A** Western blot analyzing DERL2 expression in a group of CHOL cells and HiBECs. **B** Western blot analyzing DERL2 expression in RBE and HCCC910 cells when transfected with HA-*DERL2* vectors or not. **C** CCK8 assays analyzing the HCCC910 cell proliferation when DERL2 overexpression or not. **D** CCK8 assays analyzing the RBE cell proliferation when DERL2 overexpression or not. **E** Colony formation assays analyzing the HCCC910 cell colony formation rate when DERL2 overexpression or not. **F** Colony formation assays analyzing the RBE cell proliferation when DERL2 overexpression or not. Significance: *p<0.05, **p < 0.01

Before functional assays, we investigated the expression pattern of *DERL2* in CHOL using genomics data obtained from CCLE. Analysis of the CCLE data revealed distinct expression profiles of *DERL2* in extrahepatic (shown in red) and intrahepatic (shown in black) CHOL cells (Fig. 7). Notably, the CHOL cell lines RBE, QBC939, HUCCT1, and HCCC9810 exhibited relatively higher levels of DERL2 protein expression compared to HiBEC cells (Fig. 8A).

To elucidate the specific role of DERL2 in CHOL cell growth, we introduced *DERL2*-HA vectors into RBE and HCCC9810 cells, and subsequently assessed the ectopic expression of the DERL2 protein through Western blot analysis (Fig. 8B). Overexpression of DERL2 resulted in a significant increase in cell proliferation (Fig. 8C–D), accompanied by a marked enhancement in colony formation capacity (Fig. 8E–F).

**Fig. 9:**
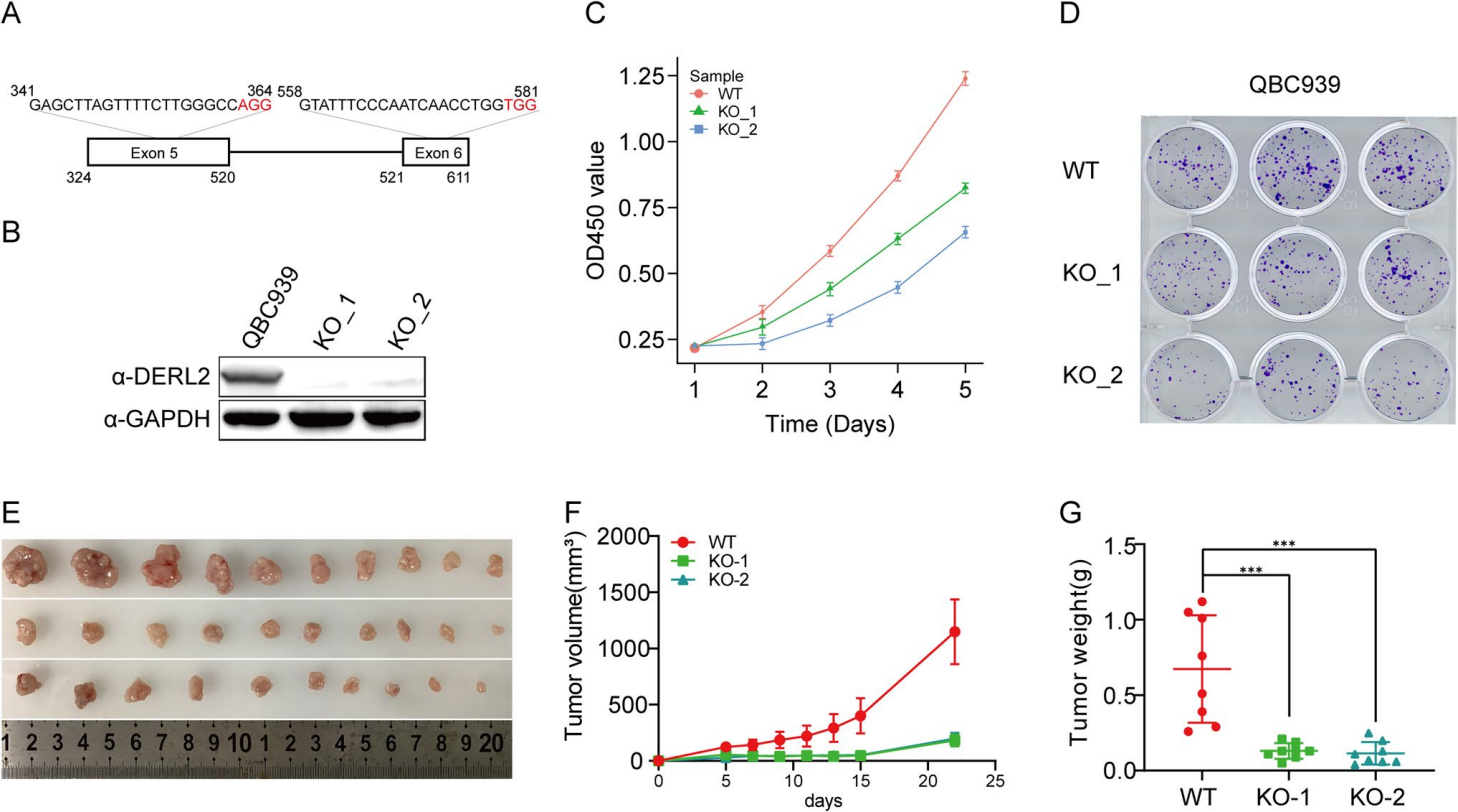
DERL2 suppresses the CHOL cell proliferation. **A** Schematic of CRISPR-Cas9 targeting design to deplete the promoter of the Exon5 and Exon6 of the *DERL2* transcript. **B** Western blot was performed on QBC939 cell post-transfection with empty vector CRISPR or CRISPR sgRNA#1 and CRISPR sgRNA#2. **C** The cell proliferation of QBC939 cells was measured by CCK8 assays when *DERL2* knockdown or not. **D** The number of colony formations of QBC939 cells was quantified by colony formation assays and then plotted when *DERL2* knockout or not. **E** Xenograft tumors induced by subcutaneous administration of two *DERL2*-KO QBC939 cells or Wild-type (WT) cells into nude mice. **F** Tumor volume of mice recorded every 5 days. **G** Tumor weight after the mice were sacrificed after 25 days. Significance: ***p < 0.001

To further validate the impact of DERL2 on CHOL cell proliferation, we employed a CRISPR/Cas9-based *DERL2* knockout system. Targeting *DERL2* Exon5 and Exon6, we successfully generated *DERL2* gene knockout QBC939 cells (Fig. 9A–B), confirmed by Western blot analysis (Fig. 9C). Depletion of *DERL2* led to a significant reduction in the colony formation rate of QBC939 cells (Fig. 9D). Consistent with the in vitro findings, mice transplanted with *DERL2-*deficient QBC939 cells exhibited a substantial decrease in tumor weight and size (Fig. 9E–G), providing further evidence of the crucial role played by DERL2 in CHOL tumorigenesis. DERL2 deficiency induces apoptosis and suppresses cell cycle transition.

**Fig. 10:**
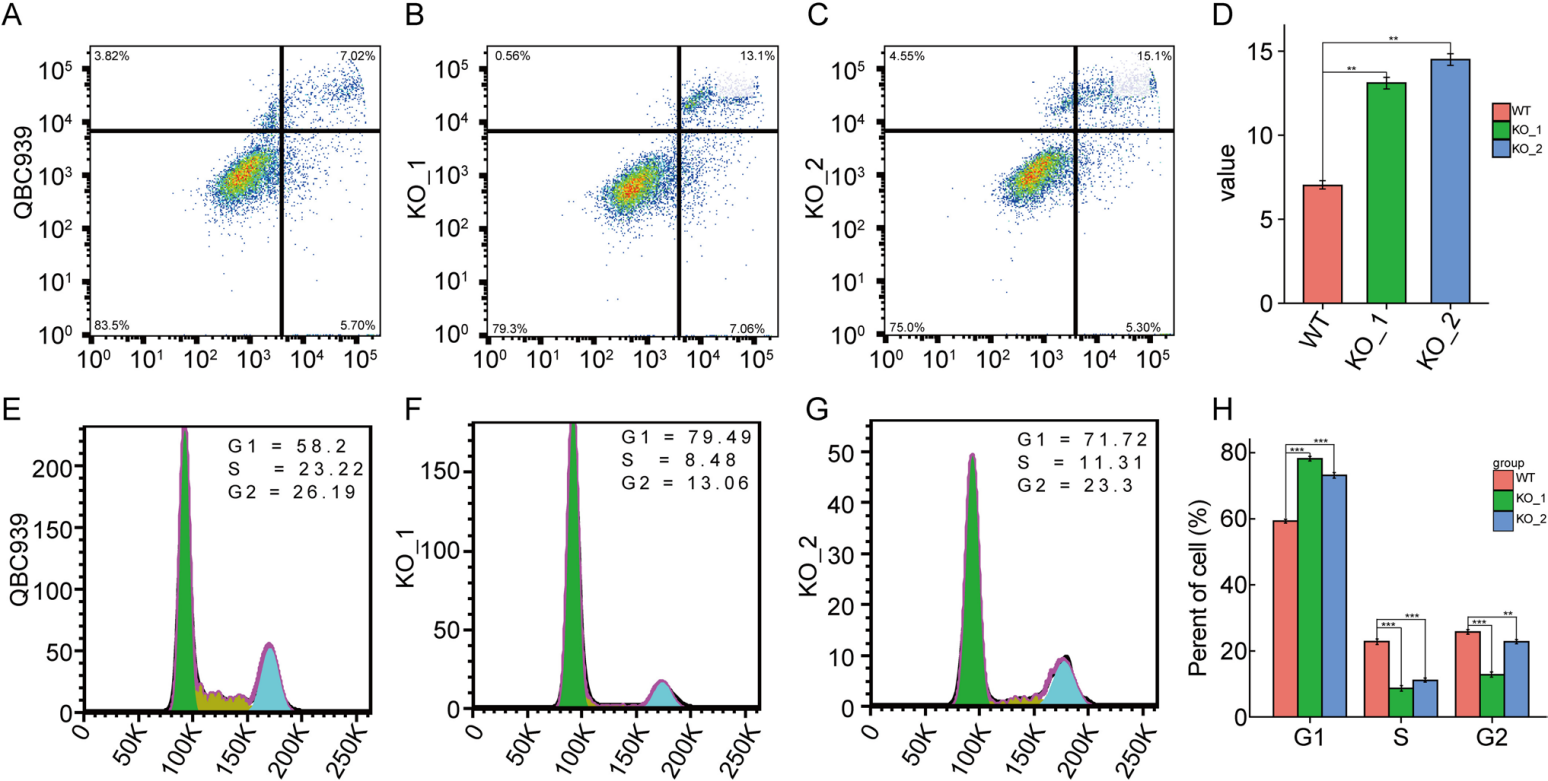
*DERL2* deficiency induces apoptosis and suppresses cell cycle transition. **A** Cell apoptosis analysis following the depletion of *DERL2* in QBC939 cells. **B** Cell cycle analysis following the depletion of *DERL2* in QBC939 cells. Significance: **p < 0.01, ***p < 0.001

Notably, the depletion of *DERL2* in QBC939 cells resulted in a significant increase in cell apoptosis (Fig. 10A). Furthermore, consistent with these findings, *DERL2*-deficient QBC939 cells exhibited cell-cycle arrest at the S and G2 phases (Fig. 10B). These observed phenotypic changes strongly suggest the significance of the DERL2 gene in CHOL progression.

### DERL2 interacting with BAG6

**Fig. 11:**
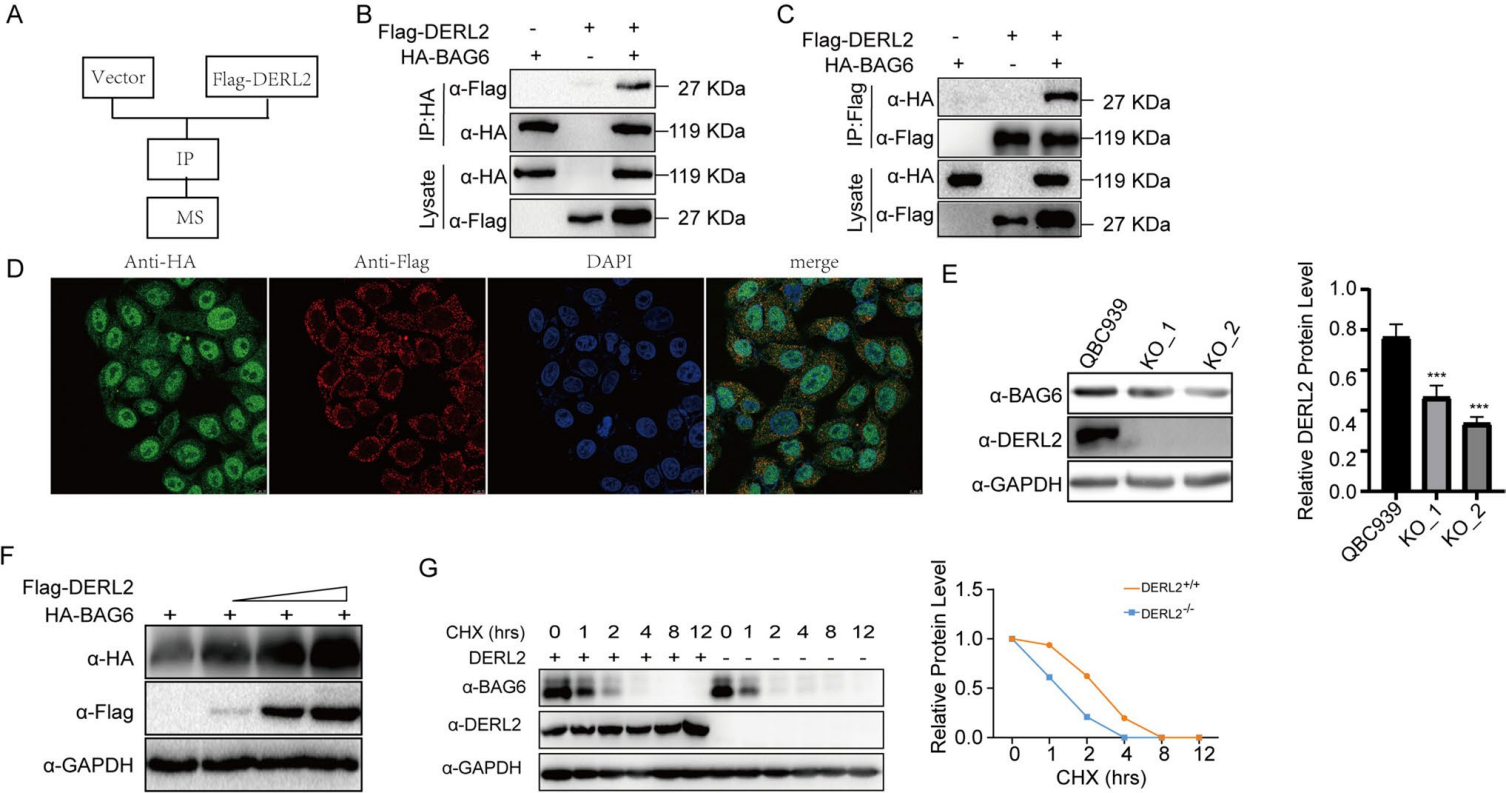
DERL2 interacting with BAG6. **A** Schematic flow chart of the experimental design. **B** 293T cells were transfected with the Flag-DERL2 or/and HA-BAG6 vectors. Forty-eight hours later, the immunoprecipitated complex with Anti-HA beads was analyzed using the corresponding antibodies. **C** 293T cells were transfected with the Flag-*DERL2* or/and HA-*BAG6* vectors. Forty-eight hours later, the immunoprecipitated complex with anti-Flag beads was analyzed using the corresponding antibodies. **D** Fluorescence confocal microscopy analysis of the colocalization of DERL2 with BAG6 in the QBC939 cells transfected with. Flag-*DERL2* or/and HA-*BAG6* vectors. **E** Western blot analysis of BAG6 expression in the QBC939 cells upon *DERL2* depletion or not. Significance: ***p < 0.001. **F** Western blot analysis of BAG6 expression in the QBC939 cells transfected the increased dose of Flag-*DERL2* vectors. **G** Western blot analysis of BAG6 expression in the BAG6-overexpressing QBC939 cells transfected with Flag-*DERL2* vectors or not. Forty-eight hours later, Chx was added. At different times later, Flag/HA antibodies were used to detect the DERL2 or BAG6 expression

**Fig. 12:**
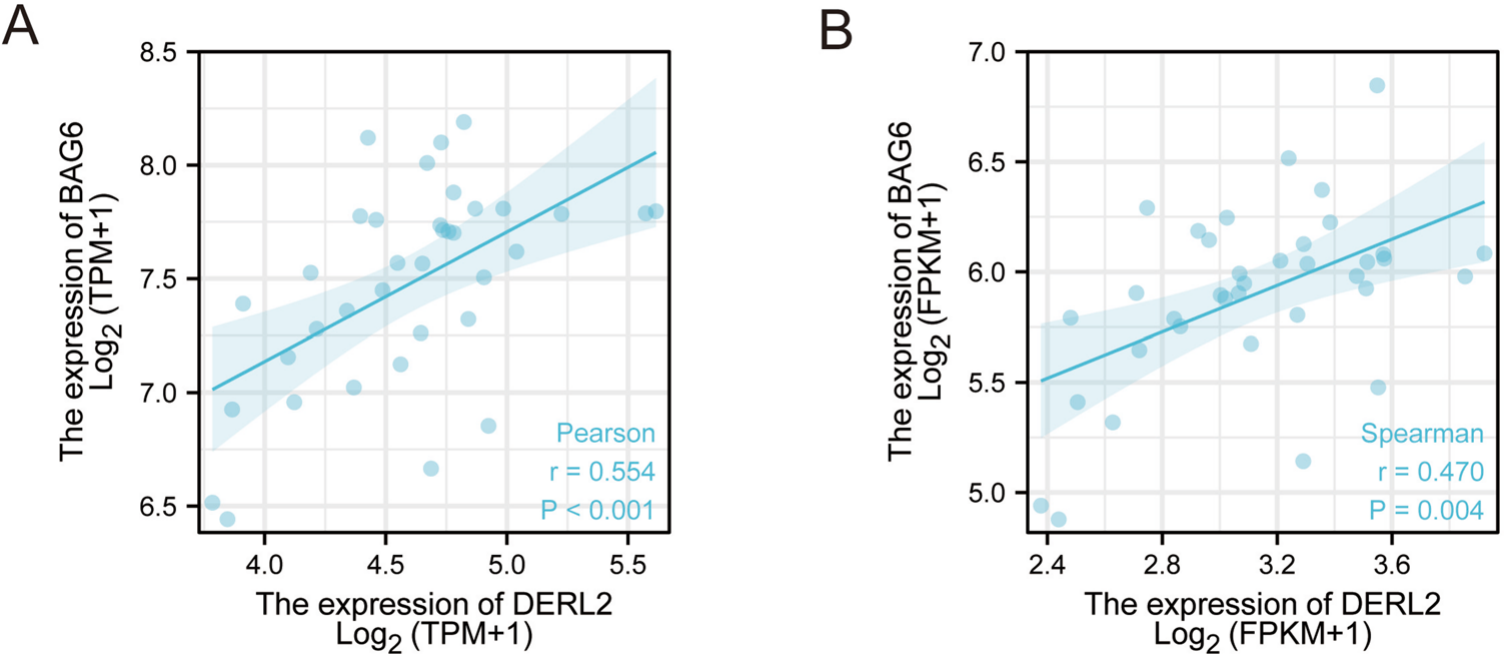
GEPIA analysis of the correlation of DERL2 with BAG6 in CHOL. **A** GEPIA analysis of the correlation of DERL2 (TPM) and BAG6 in CHOL. **B** GEPIA analysis of the correlation of DERL2 (FPKM) and BAG6 in CHOL

To investigate the mechanistic basis of DERL2’s influence on CHOL cell proliferation, we proceeded to explore its underlying interactions. We introduced DERL2-Flag recombinant vectors or empty vectors into 293T cells, followed by co-immunoprecipitation (co-IP) experiments. Mass spectrometry analysis of the DERL2-Flag complex captured by anti-Flag antibodies revealed the immunoprecipitation of BAG6 as a potential interacting protein with DERL2 (Fig. 11A). To further confirm the interaction between DERL2 and BAG6 during CHOL progression, we performed co-IP experiments in 293T cells co-transfected with Flag-*DERL2* and HA-*BAG6* vectors. Western blot analysis using HA or Flag antibody was employed to assess the expression of HA or Flag in the transfected cell lysate. Figure 11 B and C validate the interaction between DERL2 and BAG6. Moreover, the distribution of Flag-DERL2 and HA-BAG6 in QBC939 cells was examined through immunofluorescence staining. As depicted in Fig. 11 D, both proteins exhibited colocalization in QBC939 cells. This finding was further substantiated by western blot analysis, which demonstrated that depletion of DERL2 in QBC939 cells resulted in reduced BAG6 expression (Fig. 11E). Furthermore, increasing the concentration of *Flag-DERL2* vectors in QBC939 cells transfected with *HA-BAG6* vectors led to enhanced *HA-BAG6* expression (Fig. 11F). To assess the impact between proteins, cycloheximide (CHX), a protein translation inhibitor, was employed. *HA-BAG6* vectors were transfected into 293T cells with or without *Flag-DERL2* vector, followed by western blot analysis of BAG6 expression in 293T cells treated with CHX for varying durations. As shown in Fig. 11 G, BAG6 expression gradually decreased with prolonged exposure to CHX, indicating that DERL2 influenced the half-life of BAG6. Intriguingly, the tight correlation between DERL2 and BAG6 in CHOL was further substantiated by Pearson correlation analysis using the GEPIA website (Fig. 12A, B). In summary, these findings suggest that DERL2 drives the oncogenic properties of BAG6 to promote CHOL progression.

### DERL2 silence attenuates CHOL cell chemoresistance

**Fig. 13:**
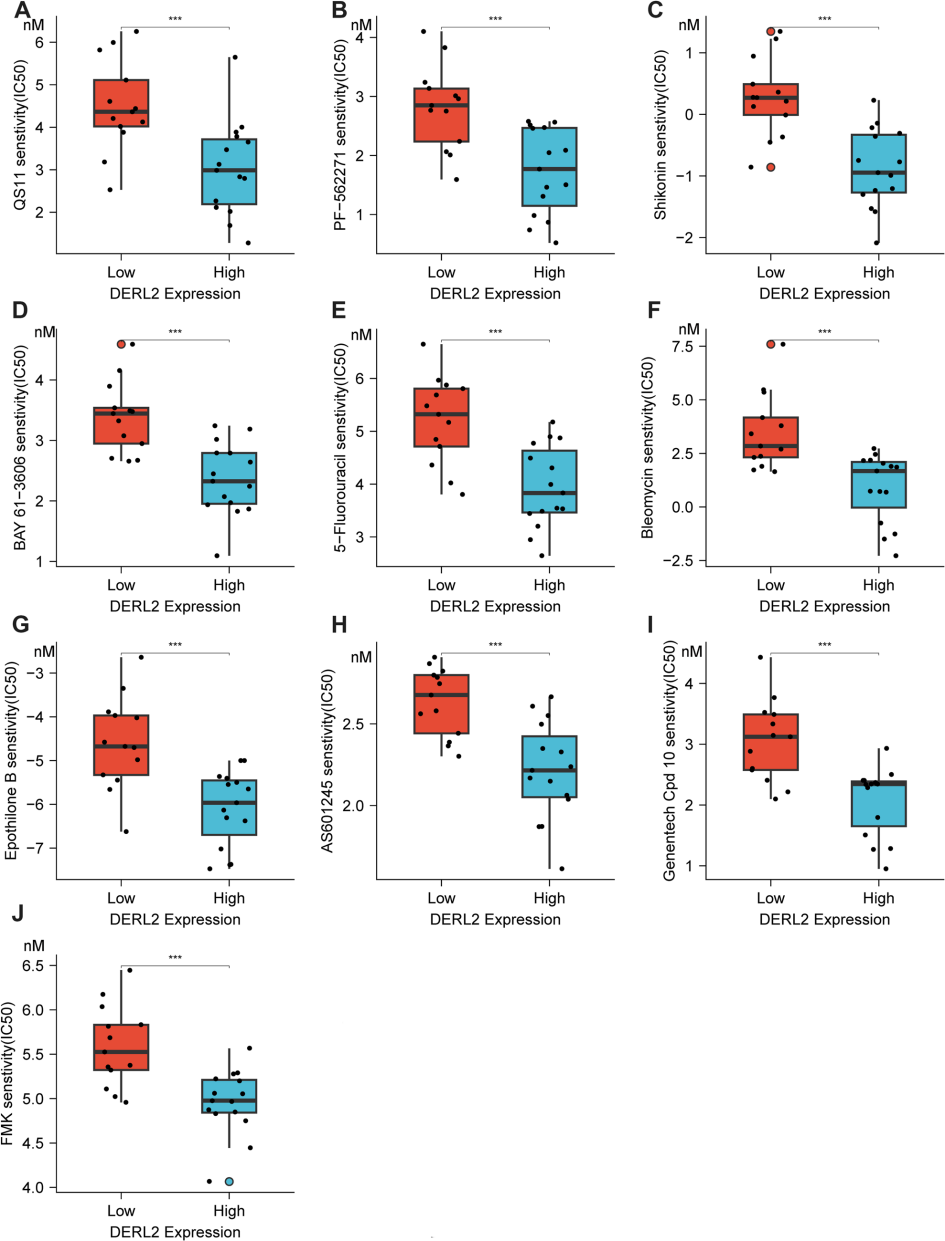
Drug sensitivity analysis in DERL2-high and DERL2-low groups using the GDSC database. Chemotherapeutic drugs included: QS11 (**A**), PF-562271 (**B**), Shikomin (**C**), BAY 61-3606 (**D**), 5-Fluorouracil (**E**), Bleomycin (**F**), Epothilone B (**G**), AS601245 (**H**), Genentech Cpd (**I**), and FMK (**J**). Significance: ***p < 0.001

**Fig. 14:**
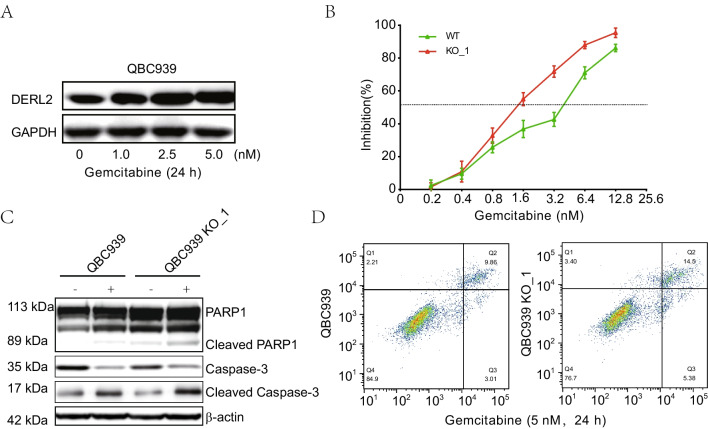
DERL2 deficiency increases the sensitivity of QBC939 cells to Gemcitabine. **A** Western blot analysis of DERL2 expression in QBC939 cells when treated with different doses of Gemcitabine. **B** The inhibition rate of Gemcitabine on QBC939 cells when DERL2 deficiency or not. Western blot analysis of the PARP1, Cleaved PARP1, caspase-3, and cleaved caspase-3PARP1 expression in DERL2-deficient and WT QBC939 cells when exposed to Gemcitabine treatment for 24h. **D** The apoptosis of QBC939 cells when DERL2 deficiency or not after Gemcitabine treatment

Previous studies have reported the chemotherapy resistance of BAG6 in breast cancer and colorectal cancer [40, 41]. To investigate the drug sensitivity of DERL2 in CHOL patients, we performed drug sensitivity analysis using the GDSC database to stratify CHOL patients based on their chemosensitivity to DERL2. Our findings revealed that *DERL2*-high CHOL patients exhibited higher sensitivity to some chemotherapeutic drugs compared to DERL2-low CHOL patients. The specific chemotherapeutic drugs included QS11, PF-562271, BAY 61-3606, 5-Fluorouracil, Bleomycin, Epothilone B, AS601245, Genentech Cpd, and FMK (Fig. 13). Additionally, other members of the Derline family have been implicated in chemoresistance in bladder cancer [42]. Gemcitabine is a standard chemotherapeutic agent used in cancer therapy. Therefore, we further investigated the impact of DERL2 expression on the sensitivity of QBC939 cells to Gemcitabine. Firstly, we assessed DERL2 expression in QBC939 cells after 24 h of exposure to different concentrations (0, 1, 2.5, and 5 nM) of Gemcitabine. Western blot analysis revealed an increase in DERL2 expression with increasing doses of Gemcitabine (Fig. 14A). Subsequently, we examined whether *DERL2* deficiency affected the sensitivity of QBC939 cells to Gemcitabine. *DERL2*-deficient QBC939 cells were treated with various concentrations of Gemcitabine (0, 1, 2.5, and 5 nM), and cell proliferation was assessed using CCK8 assays. As depicted in Fig. 14 B, *DERL2* depletion enhanced the inhibition rate of Gemcitabine on QBC939 cell proliferation. Furthermore, we evaluated key apoptotic effector molecules, such as PARP1, cleaved PARP1, caspase-3, and cleaved caspase-3, and observed that DERL2 depletion increased the expression of cleaved PARP1 and cleaved caspase-3. Moreover, this increment was further potentiated by additional Gemcitabine treatment (Fig. 14C). Importantly, after 5 nM Gemcitabine treatment, the apoptosis of DERL2-deficient QBC939 cells was also augmented compared to normal QBC939 cells (Fig. 14C). Collectively, these findings suggest that DERL2 plays a role in determining the sensitivity of CHOL to Gemcitabine.

## Discussion

Herein, using the different bioinformatic analyses, we found the highly expressed *DERL2* in CHOL progression and its tight association with worse clinical outcomes of CHOL patients. Furthermore, DERL2 was associated with several oncogenic signaling pathways. The results also showed that the highly expressed *DERL2* might favor the tumor immune infiltrates of CHOL cells. Mechanically, we found that DERL2 might interact with BAG6 and stabilize the BAG6 expression, which finally promoted the CHOL cell proliferation. Additionally, highly expressed *DERL2* induced chemotherapy resistance to Gemcitabine in the CHOL cells. Our findings suggest targeting DERL2 might effectively interfere with the CHOL progression.

Cancer cells rely on favorable endoplasmic reticulum (ER) stress conditions for their survival. However, excessive ER stress can trigger apoptosis in these cells [21, 43, 44]. To counterbalance this stress, certain cells employ the ER-associated protein degradation (ERAD) mechanism, which facilitates the clearance of misfolded and/or mislocalized proteins, including glycoproteins (ERAD substrates) within the ER lumen [17]. DERL2 has been identified as a crucial component of the ER-resident dislocation complex responsible for degrading misfolded glycoproteins in the ER. Notably, a previous investigation in chronic lymphocytic leukemia mice examined the distinct expression patterns of *DERL2* in cancerous tissues and cells. In the present study, we made novel observations regarding the differential expression of *DERL2* mRNA across various cancer types, accompanied by the presence of *DERL2* mutants in different cancers. In the case of CHOL, we detected elevated *DERL2* expression in cancerous tissues, which exhibited a strong correlation with poor clinical outcomes. Patients with high *DERL2* expression may derive potential benefits from immunotherapy and displayed strong chemoresistance to conventional chemotherapy. In in vitro cell functional assays, we provide compelling evidence that DERL2 overexpression promotes cell proliferation, whereas its knockout yields the opposite effect. Moreover, our in vitro experiments assessing cellular chemoresistance demonstrate that silencing DERL2 enhances the sensitivity of CHOL cells to Gemcitabine. Importantly, it is worth noting that other members of the Derlin family have also been implicated in modulating sensitivity to chemotherapeutic drugs [42]. Collectively, our data underscore the pivotal role of DERL2 in driving CHOL cell proliferation and chemoresistance in vitro.

BAG6, a member of the BAG gene family, exhibits widespread expression in various tissues including the testis, spleen, and 25 other tissues. Originally identified within the human major histocompatibility complex class III domain [45–47], the BAG6 gene encodes a nuclear protein that plays a significant role in cell apoptosis and autophagy [48]. Furthermore, the BAG6 complex, in conjunction with the E1A binding protein p300, assumes a critical role in the acetylation of p53 or forkhead box protein O1 (FoxO1) in response to DNA damage [49]. This complex, formed by BAG6 and a co-chaperone, facilitates the biogenesis and quality control of hydrophobic proteins [50]. Notably, BAG6 has been implicated in driving colorectal cancer progression as a nucleocytoplasmic shuttling protein [45, 51]. In a study by Ragimbeau et al., the silencing of BAG6 was demonstrated to disrupt the phospho-ubiquitylation process of mitochondrial proteins, thereby inhibiting cancer progression [45]. However, the precise identity of the critical BAG6 modulators in cancer progression remains elusive. In our current investigation, we have discovered that DERL2 functions to stabilize BAG6, potentially contributing to cancer progression.

Conclusively, our finding for the first time demonstrated the oncogenic function of robustly expressed DERL2 during CHOL progression. Furthermore, DERL2 interacted with BAG6 to favor the drug resistance of CHOL cells. Therefore, blocking the DERL2/BAG6 axis might have a strong rationale for therapies against CHOL progression.

## Declarations

The study was approved by the institutional ethics Committee of Hainan Medical University (No. GKJ190015).
